# The characterization of DNA methylation-mediated regulation of bovine placental lactogen and bovine prolactin-related protein-1 genes

**DOI:** 10.1186/1471-2199-10-19

**Published:** 2009-03-05

**Authors:** Yuki Nakaya, Keiichiro Kizaki, Toru Takahashi, Osman V Patel, Kazuiyoshi Hashizume

**Affiliations:** 1 Laboratory of Veterinary Physiology, Department of Veterinary Medicine, Faculty of Agriculture, Iwate University, 3-18-8 Ueda, Morioka, Iwate 020-8550, Japan; 2 National Institute of Agrobiological Sciences, Ikenodai 2, Tsukuba, Ibaraki 305-8602, Japan; 3 Department of Biology, Grand Valley State University, College of Liberal Arts and Sciences, 212 Henry Hall 1 Campus Dr. Allendale, MI 49401, USA; 4 Laboratory of Viral Pathogenesis, Center for Emerging Virus Research, Institute for Virus Research, Kyoto University, 53 Shogoin-Kawaharacho, Sakyo-ku, Kyoto 606-8507, Japan

## Abstract

**Background:**

Bovine trophoblast binucleate cells (BNC) express a plethora of molecules including bovine placental lactogen (bPL, gene name is *bCSH1*) and bovine prolactin-related protein-1 (*bPRP1*). *BCSH1 *and *bPRP1 *are members of the growth hormone (GH)/prolactin (PRL) gene family, which are expressed simultaneously in BNC and are central to placentation and the progression of pregnancy in cattle. However, there is a paucity of information on the transcriptional regulatory mechanisms of both the *bCSH1 *and *bPRP1 *genes. Recent studies, however, have demonstrated that the expression of a number of genes is controlled by the methylation status of their promoter region. In the present study, we examined the cell-type-specific epigenetic alterations of the 5'-flanking region of the *bCSH1 *and *bPRP1 *genes to gain an insight into their regulatory mechanisms.

**Results:**

Analysis of 5-aza-2'-deoxycytidine treatment demonstrated that *bCSH1 *expression is moderately induced in fibroblast cultures but enhanced in BT-1 cells. Sodium bisulfite based sequencing revealed that *bCSH1 *is hypomethylated in the cotyledonary tissue but not in the fetal skin, and this pattern was not altered with the progression of pregnancy. On the other hand, the methylation status of *bPRP1 *was similar between the cotyledon and fetal skin. The *bPRP1 *gene was exclusively hypermethylated in a bovine trophoblast cell-derived BT-1 cell-line. While the activity of *bCSH1 *was similar in both BT-1 and bovine fibroblast cells, that of *bPRP1 *was specific to BT-1. Treatment with a demethylating agent and luciferase assays provided in vitro evidence of the positive regulation of *bCSH1 *but not *bPRP1*.

**Conclusion:**

This is the first report to identify the differential regulatory mechanisms of the *bCSH1 *and *bPRP1 *genes and indicates that *bCSH1 *might potentially be the only transcript that is subject to DNA methyltransferase regulation. The data indicates the possibility of novel kinetics of induction of the synchronously expressed BNC-specific *bCSH1 *and *bPRP1 *transcripts, which may aid the understanding of the intricate regulation and specific role(s) of these important molecules in bovine placentogenesis and the progression of pregnancy.

## Background

Bovine placental lactogen (bPL, gene name: *bCSH1*) and bovine prolactin-related protein 1 (bPRP1) are members of the growth hormone (GH)/prolactin (PRL) family [1,2]. The homology between *bCSH1 *and *bPRP1 *is 62% for nucleotides and 36% for amino acids [1]. Bovine PL is classified as a classical member of the GH/PRL gene family [3]. In contrast, thirteen isoforms of bPRP have been identified and are categorized as nonclassical members of this family [4-7]. *BCSH1 *has been detected in various mammalian species including humans [1,8-13], while orthologues of PRP1 have been detected in several animals (e.g., rodent, cattle, goats, and sheep) [2,4-7,14-16]. In the ruminant placenta, *bCSH1 *and *bPRP1 *are concurrently detected in trophoblastic binucleate cells (BNC) [17,18]. The expression of both transcripts becomes apparent in BNC from about day 20 of gestation. Coincidentally, the earliest detection of the transcripts parallels the appearance of BNC in the fetal trophoblast, and studies indicate that *bCSH1 *is a reliable marker of BNC morphogenesis [17,18]. However, the temporal profile of these two genes are disparate during gestation such that the expression level of *bCSH1 *increases with the progression of pregnancy; whereas, the *bPRP1 *expression pattern is biphasic with a gradual increase up to mid-gestation followed by a period of steady decline [19,20]. Despite advances in the unraveling of GH/PRL gene sequences, structural characteristics, expression sites, and cell localization, little is known about their molecular regulatory mechanisms in bovines. Recent studies have revealed the transcriptional regulatory factors of the PL-family in other species; for example, Sp1 has been identified as an important factor for the expression of human *PL*; mouse *PL-I *has been shown to be activated by activator protein (AP) -1, GATA2, and GATA3; rat *PL-II *is reported to have binding sites for Ets and AP-1; and ovine *PL *is documented to be activated by AP-2 and GATA [21-25]. However, there is limited information on the factors that regulate the expression of the PRP gene, particularly in the artiodactyl species [15,16]. Recently, we reported that the AP-2 family may be involved in the regulation of these genes in bovine species [26], although additional studies are necessary to confirm this finding. However, a growing body of evidence suggests that mechanisms involving epigenetic changes regulate the expression of some genes [27-34]. Epigenetic modification involving DNA methylation has been demonstrated to be central to tissue-dependent gene expression, embryogenesis, and carcinogenesis [27-34]. We hypothesized that the differential expression patterns of *bCSH1 *and *bPRP1 *are controlled by DNA methylation, and in this study we investigated whether DNA methylation regulates the expression of the *bCSH1 *and *bPRP1*genes in trophoblast cells.

## Results

### The effects of 5-aza-dC on *bCSH1 *and *bPRP1 *expression in cultured cells

**Figure 1:**
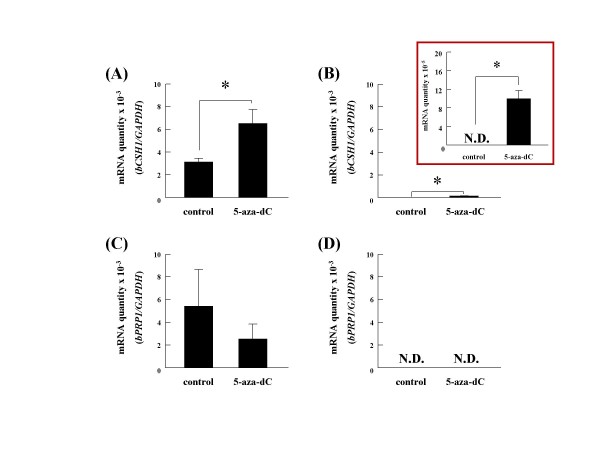
**The effects of 5-aza-dC on BT-1 and endometrial fibroblast cells**. The expression levels of *bCSH1 *(A, B) and *bPRP1 *(C, D) mRNA were determined by quantitative real-time RT-PCR. Ten μM of 5-aza-dC were added to both cell populations (A, C: BT-1; B, D: endometrial fibroblast cells). The expression levels were normalized to that of *GAPDH *mRNA. All results are displayed on a y-scale of 10^-3^, except for the insert in (B) where it is reduced to 10^-5 ^to depict the low expression levels. Values are shown as the mean ± SEM, and values marked with an asterisk are significantly different (*P < 0.05).

The *bCSH1 *expression was moderately induced in 5-aza-dC-supplemented fibroblast cultures (P < 0.05); whereas, there was an increase in the overall expression intensity in BT-1 5-aza-dC treated cultures (P < 0.05) (Figure 1A and 1B). In contrast to *bCSH1*, *bPRP1 *expression was decreased in BT-1 cells, but no *bPRP1 *expression was detected in fibroblasts (Figure 1C and 1D).

### Determination of CpG sites in the 5'-flanking region of *bCSH1 *and *bPRP1*

**Figure 2:**
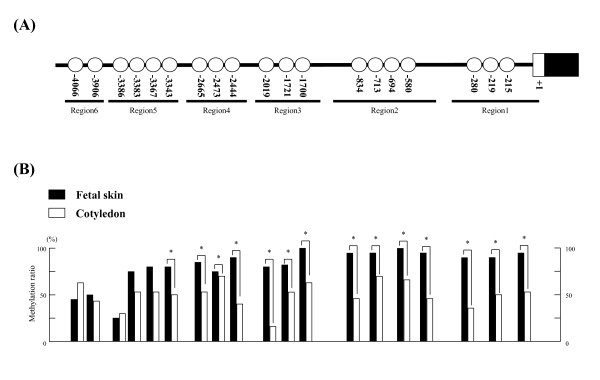
**The detection of CpG sites and the methylation status of *bCSH1 *in placentomes on day 150 of gestation**. (A) The location of CpG sites in the *bCSH1 *5'-flanking region. (B) The DNA methylation ratio of Day 150 COT (n = 3, 10 clones for each sample) and Day 150 SKIN samples (n = 2, 15 clones for each sample). The black bars indicate SKIN and the white bars depict COT samples. Values show the percentage total from 30 clones, and values marked with an asterisk(s) are significantly different (*P < 0.05).

**Figure 3:**
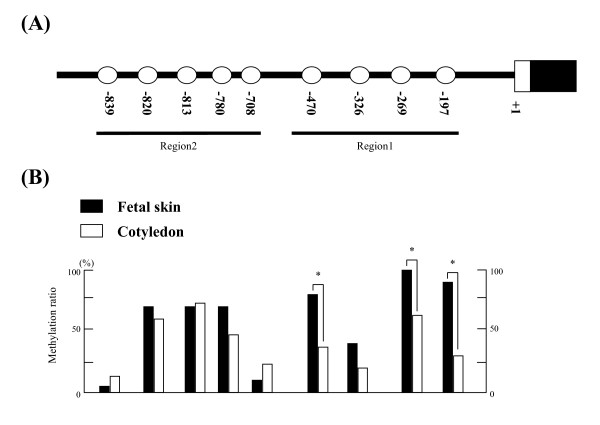
**The detection of CpG sites and the methylation status of *bPRP1 *in placentomes on day 150 of gestation**. (A) The location of CpG sites in the *bCSH1 *5'-flanking region. (B) The DNA methylation ratio of Day 150 COT (n = 3, 10 clones for each sample) and Day 150 SKIN samples (n = 2, 15 clones for each sample). The black bars indicate SKIN and the white bars depict COT samples. Values show the percentage total from 30 clones, and values marked with an asterisk(s) are significantly different (*P < 0.05).

The CpG sites in the 5'-flanking region of *bCSH1 *and *bPRP1 *were examined using Day 150 COT. In all, 19 CpG sites were detected in *bCSH1*, from -4090 to the transcription starting site, as is shown in Figure 2A. *BPRP1 *had 9 CpG sites from -860 to the transcription starting site, as is shown in Figure 3A.

### The DNA methylation statuses of cotyledon and fetal skin

Using samples from Day 150 COT and fetal skin (SKIN), the DNA methylation status of the 5'-flanking region of the *bCSH1 *and *bPRP1 *genes was examined using bisulfate sequencing.

#### bCSH1

We divided the 5'-flanking region of *bCSH1 *into 6 regions (region 1: -354 to -147 bp; region 2: -992 to -541 bp; region 3: -2099 to -1651 bp; region 4: -2711 to -2300 bp; region 5: -3428 to -3103 bp; region 6: -4094 to -3742 bp) depending on the distribution of the CpG sites. DNA was hypomethylated in the COT but not in the SKIN samples. In all, 14 CpG sites (among -3343 to -215 bp) showed a significantly low methylation status (P < 0.05). Seven of these 14 CpG sites were assumed to be located between regions 1 and 2 (Figure 2B).

#### bPRP1

We divided the 5'-flanking region of *bPRP1 *into 2 regions (region 1: -495 to -75 bp; region 2: -963 to -535 bp). The DNA methylation status was similar between the COT and SKIN samples. Only 3 CpG sites (-470, -269 and -197 bp) in region 1 had a significantly low-level of methylation (P < 0.05) (Figure 3B).

### Dynamic changes in the *bCSH1 *and *bPRP1 *gene methylation levels in cotyledonary tissue during gestation

#### bCSH1

**Figure 4:**
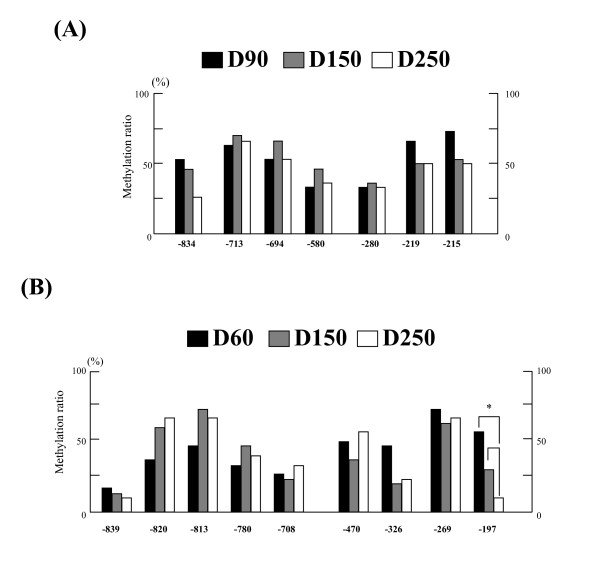
**The dynamic methylation status of *bCSH1 *and *bPRP1 *in placentomes**. (A) The dynamic DNA methylation ratios at each site in *bCSH1*. The black bar indicates Day 90 COT, the gray bar Day 150 COT, and the white bar Day 250 COT. (B) The dynamic DNA methylation ratios at each site in *bPRP1*. The black bar indicates Day 60 COT, the gray bar Day 150 COT, and the white bar Day 250 COT. Values show the percentage total from 30 clones (n = 3, 10 clones for each sample), and values marked with an asterisk(s) are significantly different (*P < 0.05).

We selected 7 CpG sites in regions 1 and 2 to compare their methylation status during gestation (Figure 4A) based on our findings of the CpG sites that exhibited significantly low methylation levels in the day 150 COT samples, as shown in Figure 2.

On day 90 of gestation, the methylation ratios at two CpG sites (-580 and -280) were lower (33.3%) in comparison to the other sites (53.3% to 73.3%). These lower ratios were maintained at the above loci throughout gestation. However, no results were significantly different (P > 0.05).

#### bPRP1

We examined a total of 9 CpG sites in regions 1 and 2 during gestation (Figure 4B). On day 60 of gestation, 7 out of the 9 sites showed a relatively low methylation ratio (< 50%). In particular, two CpG sites (-839 and -708) showed extremely low methylation ratios, and the observed ratio was maintained up to day 250 of gestation at these sites. The methylation levels increased at sites -820 and -813 by day 150, and the hypermethylation status was maintained up to day 250 at these sites. The methylation ratios at sites -326 and -197 decreased to half the values observed on day 60. One specific site, -197, exhibited a very low methylation ratio (only 10%) on day 250 of gestation (P < 0.05). A steady pattern of hypomethylation was observed at CpG sites -839, -780, and -708 throughout gestation.

### The DNA methylation status of *bCSH1 *and *bPRP1 *in BT-1

#### bCSH1

**Figure 5:**
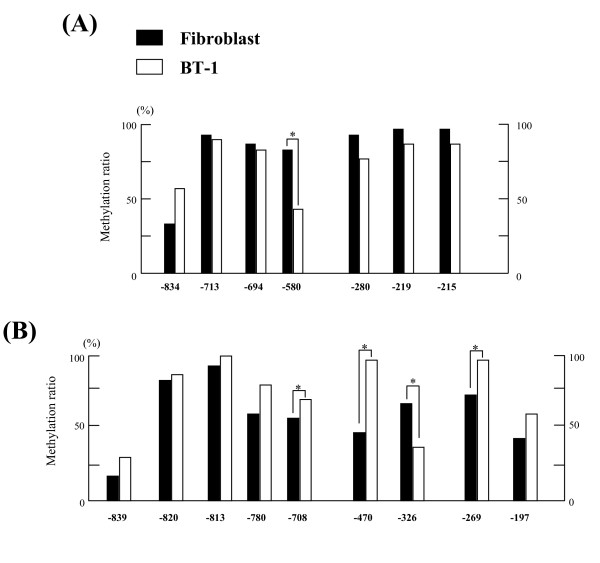
**The DNA methylation status of *bCSH1 *and *bPRP1 *in BT-1 cells**. The DNA methylation ratios of BT-1 and cotyledonary fibroblast cells in (A) *bCSH1 *and (B) *bPRP1*. The black bar indicates cotyledonary fibroblast cells, and the white bar depicts BT-1 cells. The values show the percentage total from 30 clones, and values marked with an asterisk(s) are significantly different (*P < 0.05).

Seven CpG sites in *bCSH1 *regions 1 and 2 were analyzed by sodium bisulfate sequencing using BT-1 genomic DNA. A lower (P < 0.05) methylation ratio was found at the -580 site in BT-1 cells compared to the levels detected in bovine fibroblast cells (Figure 5A).

#### bPRP1

Nine CpG sites in *bPRP1 *regions 1 and 2 were analyzed by sodium bisulfate sequencing using BT-1 genomic DNA. Seven of the 9 CpG sites were hypermethylated in the BT-1 cells (more than 50%), and six out of the 9 sites were hypermethylated in the fibroblasts. The -708, -470, and -269 sites showed a higher (P < 0.05) methylation ratio in the BT-1 cells than in the fibroblasts. Whereas, the methylation rate at the CpG -326 site was lower (P < 0.05) in the BT-1 cells (Figure 5B).

### The transcriptional activity in the 5'-flanking region of the *bCSH1 *and *bPRP1 *genes

#### bCSH1

**Figure 6:**
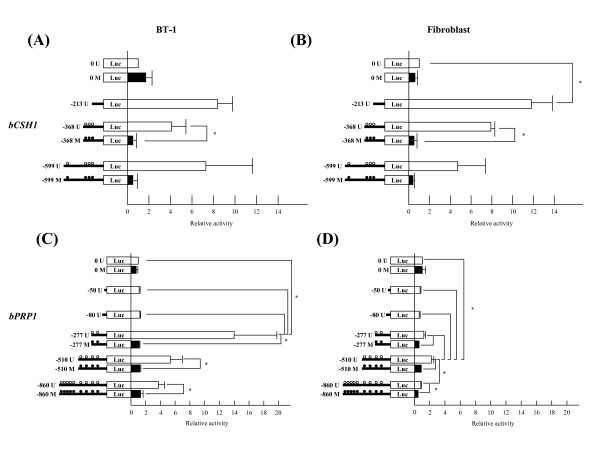
**The transcriptional activity of *bCSH1 *and *bPRP1 *constructs in BT-1 cells and fibroblasts**. The transcriptional activity of BT-1 cells (A, C) and bovine fibroblast cells (B, D). (A) and (B) show that of *bCSH1*, while (C) and (D) show that of *bPRP1*. The left figures show constructs, the horizontal lines indicate the length of the constructs, and the circles indicate the CpG sites. "M" and the black circles indicate methylated constructs, and "U" and white circles indicate unmethylated constructs. The values are shown as the mean ± SEM (n = 3), and values marked with an asterisk(s) are significantly different (*P < 0.05).

The -213 *bCSH1 *Luc construct had the highest transcriptional activity in the BT-1 (8.4 fold to the control) cells. The -368 and -599 Luc constructs had 4.1 and 7.3 fold higher activity relative to the no-promoter controls, respectively. A reduction in promoter activity was noted for the methylated -368 (P < 0.05) and -599 constructs in the BT-1 cells. The transcriptional activity of these constructs in fibroblasts was comparable: the -213, -368, and -599 constructs showed 11.8 (P < 0.05), 7.9, and 4.7 fold activity, respectively. In contrast, methylation of the -368 (P < 0.05) and -599 constructs dramatically reduced the promoter activity (Figure 6A and 6B) in the fibroblasts.

#### bPRP1

The first two fragments of the 5'-flanking region (-50 and -80) demonstrated no transcriptional activity, in BT-1 or fibroblast cells. The highest activity in the BT-1 cells was found with the -277 Luc construct (13.9 fold, P < 0.05), and increased activity was also found in the -510 (5.3 fold) and -860 (3.7 fold) constructs. Whereas, in the fibroblasts, only the -510 construct had a higher transcriptional activity than the control. Three methylated constructs from -277 to -860 displayed repressed transcription in both cell populations (P < 0.05) (Figure 6C and 6D).

## Discussion

Cell to cell interactions are crucial to the development of the placenta and the exchange of stage-specific molecular signals between the fetal and maternal units [5,6,17,18]. Specifically, these interactions are paramount to programmed fetal growth, maternal adaptation to pregnancy, and coordination of parturition. Trophoblast-specific BNC is a source of an array of biochemical products including *bCSH1 *and *bPRP1 *[17,18]. The expression profiles of *bCSH1 *and *bPRP1 *are dynamically distinct during gestation, which suggests an intrinsic regulatory role in placental formation and fetal growth in cattle [17-19]. Although, *bCSH1 *and *bPRP1 *stem from the same BNC, the mechanism by which endogenous mediators regulate the transcription and translation of these transcripts remains to be established [17]. The stage-specific differential gene expression may be directly or indirectly regulated by transcription factors [26]; however, recent studies have established that epigenetic regulation, particularly DNA methylation, is an important mode of control [27-34]. Here, we show evidence of epigenetic regulation of the *bCSH1 *transcript in bovine trophoblasts.

Treatment of bovine fibroblasts with 5-aza-dC induced an increase in the level of *bCSH1 *expression in parallel with a decrease in the expression of *bPRP1*. Ectopic expression of *bCSH1 *in endometrial fibroblasts and subsequent sequence analysis following 5-aza-dC supplementation provided proof of the concept as well as the practicality of the technique, as has been described for other genes [27-34]. Interestingly, the expression of *bCSH1 *was restricted to BNC and could not be induced in fibroblasts, including COT fibroblasts (data not shown), implying that the source of the cells as well as the cell microenvironment influences mRNA expression. Additionally, the impact of the length and position of methylation zones may be unique for each gene, and the influence of 5-aza-dC may be quenched depending on the cell/gene milieu. In contrast, compelling evidence obtained over the past decade has demonstrated that histone acetylation is linked to transcriptional activation [30]. Studies involving the *Nanog *gene, which is a key factor in maintaining the pluripotency of stem cells, revealed that its expression is not affected by 5-aza-dC; however, a combination treatment including trichostatin A facilitated expression [30]. This indicates that gene transcription is intricately regulated by a functional link between DNA methylation and histone acetylation. In this study, we did not examine the biological role of histone acetylation in relation to *bCSH1 *and *bPRP1 *regulation, and this remains to be clarified. Although the precise regulation of *bCSH1 *and *bPRP1 *remains obscure, our study provides the first evidence of a methylation-based regulatory mechanism for bovine PL-related transcripts.

We envisioned that a number of CpG islands in the 5'-flanking region of *bCSH1 *will be hypomethylated in the COT, taking into account that the *bCSH1 *gene is a trophoblast-specific transcript [17]. On the other hand, we predicted that the majority of CpG sites associated with *bCSH1 *in somatic cells, such as SKIN will likely be hypermethylated. The bisulfite sequencing confirmed the above notion, particularly, the results from the day 150 samples (Figure 2B). We further demonstrated that the methylation status of *bPRP1 *is distinct from that of *bCSH1*. Generally, the methylation pattern of both genes mirrored their expression profiles throughout gestation, particularly at the -197 site of *bPRP1*. However, it remains to be elucidated whether the changes observed in the methylation levels of both genes are to some extent dependant on the proportion of somatic versus trophoblast cells in placentomes throughout the various stages of gestation. Hence, it is important to delineate the stage-specific regulatory role of methylation patterns in relation to cell populations to facilitate interpretations of the activation/suppression of trophoblast-specific transcripts. On the other hand, we only detected three CpG sites of *bPRP1 *that were significantly different between the COT and SKIN samples at day 150 (Figure 3B). The biological significance of the variation in the methylation levels observed between the fibroblast cells and SKIN at day 150 is not clear. However, these differences could be attributable to their divergent expression patterns during gestation [5]. Additionally, the evidence from in-vitro cultures of HeLa cells indicates that the DNA methylation pattern undulates during the cell cycle of a mature somatic cell [35]. Therefore, the likelihood of some dynamic alterations in global methylation levels with progressive subcultures of fibroblasts needs verification.

During gestation, *bCSH1 *expression continuously increases with the progression of gestation, while *bPRP1 *also increases up to mid-gestation followed by a steady decline [19,20]. The question is what controls this disparate pattern of expression of these co-expressed molecules. Accordingly, we first confirmed the transcriptional activities of both genes and detected no marked difference in *bCSH1 *activation between the BT-1 cells and bovine fibroblast cells. Similarly, unmethylated *bPRP1 *constructs retained activity in both cell lines. Notably, the levels of *bPRP1 *transcriptional activity for the individual constructs were different between the fibroblasts and BT-1 cells. Also, the unmethylated *bPRP1 *constructs had lower transcriptional activity, while the methylated constructs augmented suppression. Although these results indicate that the expression of both genes is affected by their methylation status, other factors may be participating in their regulation, such as, histone acetylation or ubiquitination and/or transcription factors [27,30,36,37]. In this study, the efficiency of the transfection constructs in BT-1 cells was lower than that in the fibroblast cells (data not shown). So the efficiency of transfection may be affected by the intensity of luciferase activity, and more effective transfection methods need to be developed for precise analysis of luciferase activity. The DNA sequences of the 5'-flanking region in bovine and ovine (o)*CSH1 *are highly conserved, and the binding site of AP-2 was detected in *bCSH1 *as a consensus cis-element that is similar to the cis-element (-58 bp) that has been recognized in *oCSH1 *[3,25,38]. Furthermore, Sp1 (-220 to -215) and GATA (-475 to -470, and -105 to -99) consensus binding sites exist within the -599 region, which was confirmed by GENETYX Ver.8 (Genetyx, Tokyo, Japan). These binding sites might interact with AP-2, as these transcriptional factors are documented to regulate some placental specific genes [24-26,28,39-41]. To date, there are no reports concerning the interplay between transcriptional factors and *bPRP1*, but AP-2 may be a regulatory factor since its expression profile in bovine placenta parallels that of *bPRP1 *[26]. Ushizawa et al. [26] reported two AP-2 consensus binding sites in the upstream region within -200 (-44 to -33 and -74 to -63), but surprisingly in this study, the constructs containing these two sites showed no transcriptional activity in either the BT-1 or fibroblast cells. Therefore, the role of the above sites in transcription regulation needs further elucidation [26]. In the present study, we detected a new AP-2 consensus binding site (-267 to -260) using GENETYX Ver.8, and the constructs containing these sites, -860, -510, and -277, exhibited high *bPRP1*transcriptional activity in BT-1 cells. Therefore, it is plausible that AP-2 is involved in the transcriptional regulation of *bPRP1*. Additionally, Sp1 (-270 to -265) and GATA (-84 to -79) consensus binding sites were also detected in the upstream region of *bPRP1 *by GENETYX Ver.8 analysis. This likely denotes combinatorial regulation of the transcriptional activation and/or suppression of *bPRP1*.

In summary, our findings demonstrate that DNA methylation may regulate dynamic changes in gene expression during BNC morphogenesis, placental formation, and trophoblast differentiation in bovine species. However, further studies are necessary to understand the precise transcriptional regulatory mechanisms of *bCSH1 *and bPRP to comprehend their role in placentogenesis, fetogenesis, maternal recognition and adjustment to pregnancy, and coordination of parturition. This may also provide an insight into the roles of the various homologous and orthologous members of the GH/PRL family that are detected in the placenta.

## Conclusion

Our data indicates for the first time that there are different regulatory mechanisms for the *bCSH1 *and *bPRP1 *genes and demonstrates that *bCSHI *may be subject to transcriptional regulation by DNA methylation. These data unravel the novel kinetics of the induction of the synchronously expressed BNC-specific *bCSH1 *and *bPRP1 *transcripts, which may aid the understanding of the intricate regulation and the specific role(s) of these important molecules in bovine placentogenesis and the progression of pregnancy.

## Methods

### Animals and tissue collection

Placental cotyledonary villi (COT) and fetal skin (SKIN) were collected from Japanese black cows. The artificial insemination day was designated as day 0 of gestation. The tissues collected across specific stages of gestation were designated as follows: (i) Day 60: COT samples were collected from three different cows on days 54, 64, and 65 of gestation (n = 3 animals for bisulfite sequencing of *bPRP1*); (ii) Day 90: COT samples were collected on days 87, 88, and 97 (n = 3 animals for bisulfite sequencing of *bCSH1*); (iii) Day 150: COT samples were collected on days 144 (n = 2 animals) and 150 (n = 3 animals for bisulfite sequencing), and SKIN samples were collected from both animals (n = 2 animals for bisulfite sequencing) and designated as Day 150 SKIN; and (iv) Day 250: COT samples were collected on days 245, 249, and 252 (n = 3 animals for bisulfite sequencing). The collected samples were stored at -80°C prior to DNA extraction. All procedures for these animal experiments were carried out in accordance with guidelines approved by the Animal Committee of Iwate University and the National Institute of Agrobiological Sciences for the use of animals.

### Cell culture

BT-1 cells established from in vitro blastocysts [42,43] were cultured according to a previously described method [44]. Briefly, the cells were cultured in Dulbecco's modified Eagle's medium/F-12 medium (DMEM/F-12, Sigma, Saint Louis, MI, USA) containing 100 IU/ml of penicillin and 100 μg/ml of streptomycin (Sigma) supplemented with 10% fetal bovine serum (FBS, HANA-NESCO, Tokyo, Japan) at 37°C in an atmosphere of 5% CO_2_. The medium was changed every two or three days. A monolayer of confluent BT-1 cells was mechanically passaged by pipetting. The dissociated cell clumps in the medium were plated on collagen-coated flasks. The cell clumps attached themselves to the flasks and proliferated to re-form a monolayer. Successful passages were also achieved by transferring multicellular vesicles that had spontaneously formed from the cell colony. Bovine endometrial or cotyledonary fibroblast cells, which had been derived from the uteruses of Japanese black cattle and established as detailed elsewhere [45], were cultured in DMEM/F-12 (sigma) containing 100 IU/ml of penicillin and 100 μg/ml of streptomycin (Sigma) supplemented with 10% FBS (HANA-NESCO) at 37°C in an atmosphere of 5% CO_2_. In this study, 4th passaged endometrial fibroblast cells or 9th passaged cotyledonary fibroblast cells were used. The medium was changed every two to three days. A monolayer of confluent fibroblast cells was passaged and scaled down using 0.25% Trypsin-EDTA (Sigma) and plated on flasks.

### 5-aza-2'-deoxycytidine treatment

A confluence of 30–40% of bovine endometrial fibroblast cells or cotyledonary fibroblast cells was plated on a 100-mm dish and incubated for 24 h. Then, the cells were treated with either vehicle or 10 μM 5-aza-2'-deoxycytidine (5-aza-dC) in an FBS-containing medium for 72 h. The medium and 5-aza-dC were changed every 24 h. The BT-1 cells were treated as described above.

Total RNA was isolated from the cultured cells using TRIzol Reagent (Invitrogen, Carlsbad, CA, USA) according to the manufacturer's instructions. The RNA concentration was calculated by measuring the absorbance with a spectrophotometer (U-2000A, HITACHI, Tokyo, Japan). All RNA samples were stored at -80°C prior to processing.

### RT-PCR

One microgram of total RNA was reverse transcribed into cDNA with a Random Primer (TOYOBO, Osaka, Japan) and Superscript III reverse transcriptase (Invitrogen). PCR was performed using AmpliTaq Gold DNA polymerase (Applied Biosystems, Foster City, CA, USA). The annealing temperature was 60°C, and the PCR involved 35 cycles. The PCR products were analyzed by agarose gel electrophoresis and visualized by ethidium bromide staining. The respective primer sets for *bCSH1*, *bPRP1*, and *GAPDH *are listed in Table 1. All the primers were designed using Primer 3 [46] and commercially synthesized (Greiner, Tokyo, Japan). The PCR products were extracted from agarose gel and purified using a GENECLEAN III Kit (MP Biomedicals, Solon, OH, USA). The purified PCR products were ligated to the pGEM-T Easy vector (Promega, Madison, WI, USA) and amplified in DH-5α (Invitrogen). All plasmids were purified using the QIAprep Spin Miniprep Kit (QIAGEN, Valencia, CA, USA) and sequenced using an ABI Prism 3100 automatic sequencer (Applied Biosystems).

**Table 1:** The Oligonucleotide primers used for the RT-PCR and quantitative real-time RT-PCR.

Gene	Strand	Sequence	Position
**RT-PCR**			
*bCSH1* (NM_181007)	Forward	5'-CTGCTGGTGGTGTCAAATCTAC-3'	170–191
	Reverse	5'-TGGTTGGGTTAATTGTGGGC-3'	828-812
*bPRP1* (NM_174159)	Forward	5'-CACGGTCAACAGGAGTCCTCACC-3'	43–65
	Reverse	5'-AATTTCAGGTAGCCCGCTGTGG-3'	873-852
*GAPDH* (NM_001034034)	Forward	5'-CCTTCATTGACCTTCACTACATGGTCTA-3'	173–200
	Reverse	5'-GCTGTAGCCAAATTCATTGTCGTACCA-3'	1029-1003
			
**real-time RT-PCR**			
*bCSH1*	Forward	5'-GCAACATTGGTGGCTAGCAA-3'	281–300
	Reverse	5'-GCCCTCGCCAAACTGTTTATTA-3'	358-337
*bPRP1*	Forward	5'-CACGGAGCTGCAGCATATGA-3'	501–520
	Reverse	5'-CCTTGTGGCGCTTGATAGGA-3'	558-539
*GAPDH*	Forward	5'-AAGGCCATCACCATCTTCCA-3'	280–299
	Reverse	5'-CCACCACATACTCAGCACCAGCAT-3'	355-332

### Quantitative real-time RT-PCR

Real-time RT-PCR was performed using the SYBR Green Detection System (Applied Biosystems) according to the method described previously [26]. PCR and the resulting increase in reporter fluorescent dye emission were monitored in real time using a 7300 Real Time PCR System (Applied Biosystems). The primer pair was designed by the Primer Express Program (Applied Biosystems). The primers for each gene are listed in Table 1. The thermal cycling conditions included one cycle at 50°C for 2 min, one cycle at 95°C for 10 min, and 40 cycles at 95°C for 15 s and 60°C for 1 min. To quantify the mRNA concentrations, standard curves were generated for each gene by serial dilution of the plasmids containing their cDNA. The expression ratio of each gene was normalized relative to the abundance of a validated endogenous control *GAPDH *mRNA to adjust for variations in the RT-PCR reaction. Quantitation was performed using three separately cultured samples per condition, and all values are presented as mean ± SEM.

### Genomic DNA extraction

The genomic DNA was extracted from COT, SKIN, and cultured cells using a Puregene DNA Purification System (Gentra Systems, Minnesota, U.S.A.) according to the manufacturer's instructions. The DNA concentration was calculated by a spectrophotometer. All DNA samples were stored at -30°C prior to processing.

### Preparing constructs for sequencing and luciferase reporter assays

The sequence of the 5'-flanking regions of *bCSH1 *and *bPRP1 *was confirmed by cloning them. Each fragment of the *bCSH1 *and *bPRP1 *regions was amplified using PCR with primers designed on Map Viewer, which is available on the NCBI web site [47]. Each PCR was performed using KOD-Plus-Ver.2 (TOYOBO). The primer sets used are listed in Tables 2 and 3. The annealing temperature was set at 62 to 65.5°C, and the PCR lasted 30 cycles. The PCR products were analyzed by agarose gel electrophoresis and visualized by ethidium bromide staining. The PCR products were cut by Kpn I (TOYOBO) and Xho I (TOYOBO), or Sac I (TOYOBO) and Xho I, before being cloned into the pGL3-Basic vector (Promega) using a DNA Ligation Kit Ver.2.1 (TaKaRa, Tokyo, Japan). All constructs were amplified in SCS110 (Stratagene, La Jolla, CA, USA), which lacks two methylases (dcm and dam), and sequenced using an ABI Prism 3100 automatic sequencer (Applied Biosystems) [30].

**Table 2:** The primers used for the *bCSH1 *(NW_001494181) 5'-flanking region of the sequencing and reporter constructs.

Position	Strand	Sequence	Annealing
-4090 to -4073	Forward	5'-ATGGTACCATTGTCTATTACAGGGTGCA-3'	65.5
-599 to -581	Forward	5'-GGGGTACCCCTGTCCTAGTTCTTTAAC-3'	64
-368 to -353	Forward	5'-GGGGTACCCCTTAGATCTCTGAGTAG-3'	62.6
-213 to -194	Forward	5'-GGGGTACCCCATAGGGTGTATACAGATAC-3'	62
-8 to +8	Reverse	5'-ATCTCGAGATGGGAATGCCTAAGGAG-3'	

^a ^All forward primers have a KpnI recognition site (-GGTACC-), and the reverse primer has an XhoI recognition site (-CTCGAG-) at its 5'-end. ^b ^The reverse primer is common to all PCRs.

**Table 3:** The primers used for the *bPRP1 *(AH001153) 5'-flanking region of the sequencing and reporter constructs.

Position	Strand	Sequence	Annealing
-860 to -845^a^	Forward	5'-GGGAGCTCCCTGTAAAATATCATGTA-3'	62
-510 to -495^a^	Forward	5'-GGGAGCTCCCATTAATACCAACACAG-3'	62
-277 to -262^a^	Forward	5'-GGGAGCTCCCGACTCCTCCGCCCATG-3'	62
-80 to -65^b^	Forward	5'-GGGGTACCCCAGCTCTACTCCACAGG-3'	65.5
-50 to -35^b^	Forward	5'-GGGGTACCCCTTTTATGGCCTCATGG-3'	65.5
+23 to +38^c^	Reverse	5'-ATCTCGAGATGGGAATGCCTAAGGAG-3'	

^a ^The primers have a SacI recognition site (-GAGCTC-).^b ^The primers have a KpnI recognition site (-GGTACC-). ^c ^The common reverse primers for all forward primers have an XhoI recognition site (-CTCGAG-) at their 5'-end.

### Sodium bisulfite genomic sequencing

CpG methylation status was examined at each stage of gestation (Day 90 COT, Day 150 COT, Day 250 COT, and Day 150 SKIN for *bCSH1*; Day 60 COT, Day 150 COT, Day 250 COT, and Day 150 SKIN for *bPRP1*) as well as in cultured cells (BT-1 and bovine fibroblast cells) by sodium bisulfite genomic sequencing analysis. The details of the sodium bisulfite genomic sequencing are contained in previous reports [32,33]. Briefly, DNA (1 μg) digested with EcoRI (for *bCSH1*) or BamHI (for *bPRP1*) was denatured in 0.3 M NaOH at 37°C for 15 min. Then, 3.6 M sodium bisulfite (pH 5.0) and 0.6 mM hydroquinone were added, and the sample underwent 15 cycles of 30 s denaturation at 95°C and 15 min of incubation at 50°C. The sample was desalted using the Wizard DNA Clean-up system (Promega) and desulfonated in 0.3 M NaOH. DNA was ethanol precipitated and dissolved in 40 μl of Tris-EDTA buffer. All modified DNA samples were stored at -80°C prior to processing.

The DNA fragments were amplified with bisulfite PCR using AmpliTaq Gold (Applied Biosystems) and the set of primers described in Tables 4 and 5. The primer design was performed using MethPrimer [48]. The annealing temperature was set at 56 to 62°C, and the total number of cycles was 35. The PCR products were analyzed by agarose gel electrophoresis and visualized by ethidium bromide staining. The PCR products were cloned into a pGEM-T Easy vector (Promega). Ten clones from COT and 15 clones from SKIN for each sample and region (calculated as total 30 clones per region) were sequenced using an ABI Prism 3100 automatic sequencer (Applied Biosystems), and the methylation ratios of the samples were determined. Thirty clones from cultured cells from each sample and region were sequenced using an ABI Prism 3100 automatic sequencer. All results are shown as percentages.

**Table 4:** The primers used for the bisulfite sequencing of *bCSH1*.

Region	Strand	Sequence	Annealing
Region 1	Forward	5'-AGGGAAGATTTTTTGGAGAAGG-3'	60
	Reverse	5'-AATAATAACCTTCAAATAACCAATACAC-3'	
Region 2	Forward	5'-AAGAGTTTGTATGGATTTTTTTAGA-3'	60
	Reverse	5'-CCACACTCTTCCTCAATAATAATAA-3'	
Region 3	Forward	5'-TTTGATAATTGTTTATTGAATGATTTATTA-3	60
	Reverse	5'-CAAATTCACTCCTAACCTATCTTTTCT-3'	
Region 4	Forward	5'-AGTTTGTTAATAAATGAATTTTTTTT-3'	56
	Reverse	5'-CTTACTTTTTCCTCTTTTCTACCCTAAA-3'	
Region 5	Forward	5'-GTTTGGGGTTGAATATTTATTATT-3'	60
	Reverse	5'-TACCTACTTCTATTTAATACCAATT-3'	
Region 6	Forward	5'-ATGTTGTTTATTATAGGGTGTATA-3'	56
	Reverse	5'-TTATATCTTTTACAATTTTAATACTAA-3'	

**Table 5:** The primers used for the bisulfite sequencing of *bPRP1*.

Region	Strand	Sequence	Annealing
Region 1	Forward	5'-GGGATTGTTGTTGTTGTTGTTAAGT-3'	60
	Reverse	5'-AAAACTATCTCTTTCTCCATACTAATACCT-3'	
Region 2	Forward	5'-TTGTGTATGAGATAGTAAAAGAGATATTGA-3'	60
	Reverse	5'-CAATAAAAAACCAAAAAAACTATAATTACA-3'	

### In vitro methylation of constructs

The constructs for the luciferase reporter assay were methylated *in vitro *by SssI CpG methylase (New England BioLabs) in the presence of 160 μM of S-adenosylmethionine at 37°C for 3 h [30]. Completion of the methylation reaction was confirmed when digestion with Hha I was no longer possible [49].

### Luciferase reporter assay

The constructed plasmids with or without methylation were transfected into BT-1 using ExGen 500 *in vitro *Transfection Reagent (Fermentas, Hanover, USA) and bovine fibroblast cells using FuGENE 6 (Roche Diagnostics, Basel, Switzerland). After 10 days of passaging, the BT-1 cells were transfected with 5 μg of constructs and 50 ng of pRL-TK (Promega), and cultured in the conditions detailed above for 72 h. Bovine fibroblast cells were transfected with 200 ng of constructs and 2 ng of pRL-TK and cultured in the conditions described above for 48 h. The luciferase activity was measured by a TR-717 Microplate Luminometer (Applied Biosystems) [50]. The activity of all constructs was determined using a Dual-Luciferase Reporter Assay System (Promega) according to the manufacturer's instructions. Assays were performed three times, and all results are shown as the mean ± SEM.

### Statistic analysis

The Student's t-test and Pearson's chi-square test were used to analyze the statistical differences between the 5-aza-dC supplemented and non-supplemented samples and the sodium bisulfite treated genomic sequences, respectively. Differences among luciferase reporter constructs were assessed by one-way ANOVA, followed by the Tukey-Kramer multiple comparison test. Differences were considered significant at P < 0.05.

## Authors' contributions

YN participated in the design of the study, carried out most of the experiments, and wrote the manuscript. KK participated in coordinating the design of the study. TT supplied the tissue samples. OVP participated in the discussion and provided insights into the manuscript. KH planned and participated in coordinating the design of the study, contributed to drafting the manuscript, and supervised the process. All authors have read and approved the final manuscript.
